# Perceived Corona virus exposure as a function of interpersonal distance and time of a conversation

**DOI:** 10.1007/s44155-022-00027-9

**Published:** 2022-12-05

**Authors:** Ola Svenson

**Affiliations:** 1 Stockholm University, Stockholm, Sweden; 2 Decision Research, Eugene, OR USA

**Keywords:** COVID-19, Perceived virus exposure, Interpersonal distance, Time of exposure, Risk perception, Risk communication

## Abstract

**Background:**

During the COVID-19 pandemic people were asked to keep interpersonal distance, wash their hands and avoid gatherings of people. But, do people understand how much a change of the distance to a virus infected person means for the exposure to that person’s virus? To answer this question, we studied how people perceive virus exposure from an infected person at different distances and lengths of a conversation.

**Method:**

An online questionnaire was distributed to 101 participants drawn from the general US population. Participants judged perceived virus exposure at different interpersonal distances to an infected person in a face to face conversation of different lengths of time. A model based on empirical and theoretical studies of dispersion of particles in the air was used to estimate a person’s objective virus exposure during different times and distances from a virus source. The model and empirical data show that exposure changes with the square of the distance and linearly with time.

**Results:**

A majority (78%) of the participants underestimated the effects on virus exposure following a change of interpersonal distance. The dominating bias was assuming that exposure varies linearly with distance. To illustrate, an approach to a virus source from 6 to 2 feet was judged to give a 3 times higher exposure but, objectively it is 9 times. By way of contrast, perceptions of exposure as a function of the duration of a conversation were unbiased. The COVID-19 pandemic caused by the SARS-CoV2 virus is likely to be followed by other pandemics also caused by airborne Corona or other viruses. Therefore, the results are important for administrators when designing risk communications to the general public and workers in the health care sector about social distancing and infection risks.

**Conclusions:**

People quite drastically underestimate the increase in virus exposure following an approach to a virus infected person. They also overestimate exposure after a move away from an infected person. For public health reasons, the correct function connecting distance with virus exposure should be communicated to the general public to avoid deliberate violations of recommended interpersonal distances.

**Supplementary Information:**

The online version contains supplementary material available at 10.1007/s44155-022-00027-9.

## Background

The COVID-19 pandemic caused by the SARS-CoV2 virus is likely to be followed by other pandemics also caused by airborne Corona or other viruses. During the COVID-19 pandemic people are asked to keep distance, wash their hands and avoid gatherings of people. But, do people know to what extent an interpersonal distance and length of a conversation influence virus exposure? The present study was focused on how people perceive virus exposure from an infected person at different distances and lengths of a conversation. To exemplify, what does it mean to a person's virus exposure if she or he moves closer from, e.g., 6 feet to 2 feet from a person who is infected with an airborne virus like the SARS-CoV2? What does it mean to extend a conversation from 1 to 5 min? To answer these questions, we asked participants to judge virus exposure after changes of interpersonal distance and length of a face to face conversation with a virus infected person. The judgments were related to empirical and theoretical facts concerning the spread of virus particles in the air. To the best of our knowledge, there are no studies of perceived exposure as a function of changes of interpersonal distance except the study by Svenson and colleagues [1] who found that most participants underestimated the protective effect of moving away from another person. Correspondingly, most participants were unaware of how much their exposure would increase if they moved closer to an infected person. The purpose of the present study was to validate the findings of that study and extended the scope to include other problems and the duration of a conversation.

### Droplets and aerosols

The distance that particles travel away from an infected person depends on many different factors, such as the size of the particles, initial momentum with which they are expelled (regular conversation, singing coughing etc.), position of the head and the body of the person emitting the particles, strength (velocity), structure (turbulent or laminar), direction, temperature and humidity of airflow and individual differences between people. A SARS-CoV2 virus can travel on droplets that are greater or smaller than 5 μm. Droplets that are exhaled from a person and are greater than 5 μm follow the laws of gravity and fall to the ground within some distance from the exhaling person [2]. Droplets smaller than 5 μm, called aerosols, can originate directly from an exhalation or from evaporated greater droplets and their movements follow the streams of air and can stay in the air for a long time [3–5]. The aerosols can provide ambient virus exposure. Early after an exhalation, the ambient dispersion effects can be ignored because the droplet-laden effects seem to dominate, but after some time aerosols accumulate and the ambient effect takes over and determines the exposure if a space is not properly ventilated [6]. Hence, a person’s exposure to droplets and aerosols depend not only on distance but also on other factors and situations.

**Fig. 1:**
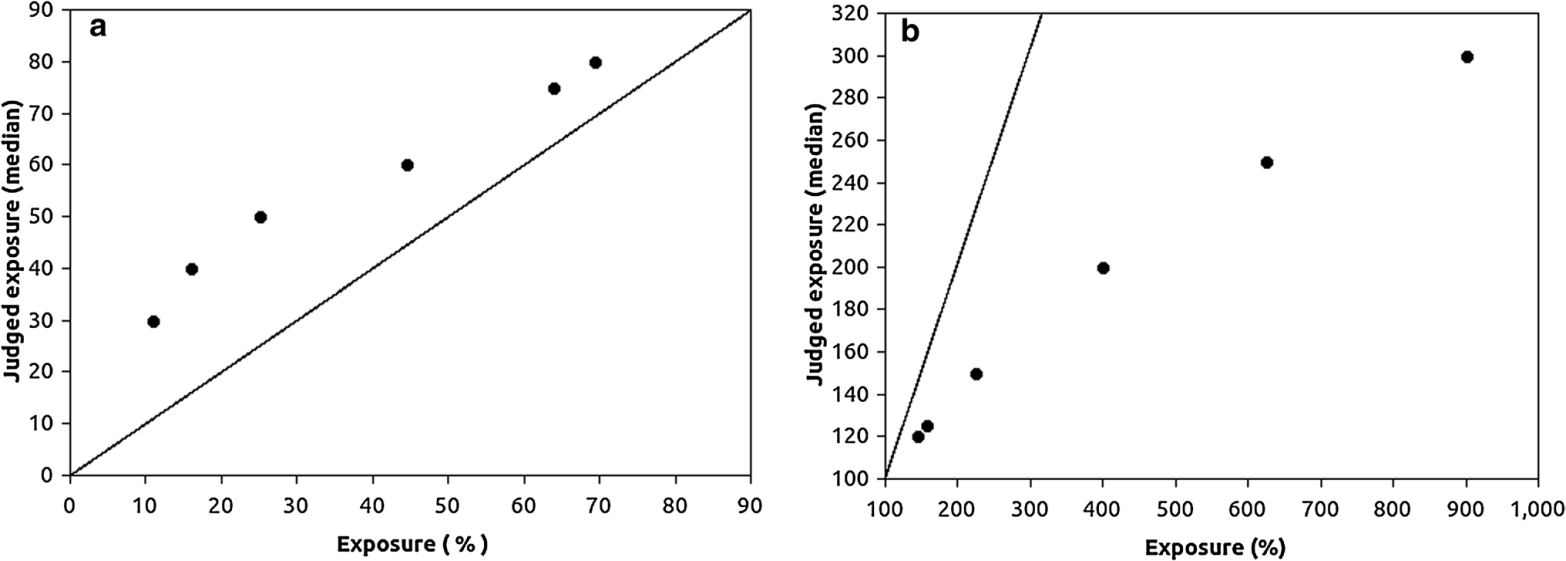
Median judged exposure in percent after having moved away from a person plotted against model predictions (**a**) and after having moved closer to a person (**b**); the straight lines describe VEM predictions

A physical theory of virus exposure that can be related to perceived exposure should be based on virus distribution in the air under different conditions. Balachandar and co-authors [7] gave an extensive overview and possible solutions for how to solve the multiphase flow problems created by droplets and aerosols carrying viruses. To illustrate, Bourouiba [8] specified how far the larger droplets but also smaller aerosols can travel after a sneeze or cough (7–8 m). In the present studies we treated only normal breathing conditions. Bjørn and Nielsen ([9], p. 155, Fig. 15) reported exposure to another person’s normal breathing in a calm laboratory face to face setting with different distances (0.4 to 1.2 m). The power function Exposure = 1.90 × Emission × Distance^−2.2^ describes their results. In another empirical study by Nielsen et al. ([10], p. 557, Fig. 8) the power function was Exposure = 4.3 × Emission × Distance^−2.3^ (0.35 to 1.10 m) with a different constant depending on different measures of relative exposure used in the different studies. Melikov [11] presented an overview of studies with exposure as a function of distance reported by different authors [12–17]. The results were summarized by Fig. 1 in Melikov [11] with a decreasing function that can be approximated by a power function of distance with an exponent that is − 2 or smaller. This was also confirmed by Wang, Xu and Huang [18]. The laboratory results presented by Nielsen and collaborators and most of the above cited researchers were obtained in conditions similar to the situation presented to the participants in the present study, a face to face conversation with no coughing or sneezing, but a change of interpersonal distance and relatively short interactions.

### Model

Based on the results of previous research, we used the power function in Eq. (1) to serve as a model for objective exposure. We set the exponent to 2.0 in the model. It is clear from the empirical studies cited above that this exponent if anything, underestimates the speed of decrease of exposure with increasing distance. This allows some margin on the conservative side when comparing subjective judgments with physical facts. In Eq. (1), Epv is exposure to virus, *a* and *n* are constants, *E* emitted virus during time *t*, and D distance to source:

1 $${\text{Epv}} \, \text{=} \, {\text{a}}*E*t*{D}^{-n} \,\quad D>0, \, t>0$$

We will call the function in Eq. (1) the Virus Exposure Model, VEM. The exponent describes change of exposure as as a function of change of distance. Applying n = 2.0 to a person who approaches another infected person from 6 to 2 feet, VEM predicts an increase of exposure to be (6/2)^2^ = 9 times. However, a person who applies a linear model, n = 1.0 will judge the exposure to be 6/2 = 3 times the initial exposure after the approach. In the following, we will use VEM to describe the model with the exponent = 2.0 if nothing else is indicated. The study by Svenson and colleagues [1] on perceived exposure showed that different judgment models and exponents were used across participants. Many participants used a linear cognitive model and a majority of the participants used models that underestimated the effects on exposure following both approach and withdrawal from another person.

Welsch and colleagues [19] asked their participants to indicate the interpersonal distance that they preferred when no virus was around and during the Covid-19 pandemic and the distances were 1.18 m and 1.83 m. However, the latter distance decreased over time during the pandemic to 1.41 m in some kind of adaptive process. Hall [20] suggested that people prefer different interpersonal distances: close distances for partner/family (up to 0.45 m), distance to friends (0.45–1.20 m) and distance to strangers (1.20 − 3.65 m). With reference to these distances and recommended interpersonal distance during the pandemic, we used distances from 2 feet (0.61 m) to 6 feet (1.83 m) in the present study. We also asked about preferred interpersonal distances to another person when no virus was around and when the other person was infected with a Corona virus. In the present study, there was always an infected person and the participants judged changes in exposure following both changes of interpersonal distance and changes in length of a conversation. Based on earlier results [1], we predicted that the effects on virus exposure from changing interpersonal distance would be underestimated for both approaching and moving away conditions. A linear function is the most easily available function when the applicability of functions from a hierarchy of subjective functions are tested in a new context [21]. Therefore, we predicted that a majority of the judgments of exposure would be described by a linear function of time and not systematically biased.

## Empirical study

### Participants

In all, 101 participants aged 18 years or more were recruited by Prolific from a general US adult English speaking population. The questionnaire was distributed and answered October 27, 2021. One participant was eliminated because of failure on an attention test and 4 participants were excluded because they did not fulfill the task by giving only zero or 100 as answers to the questions. Hence, the study included 96 participants (48 women, 47 men and 1 unspecified). The mean age was 31 (SD = 11.4) with a range from 19 to 73 years. Demographic data can be found in the supplement.

### Procedure and problems

A Qualtrics questionnaire was distributed to the participants and the instruction started with the following. “*As you probably know, the Coronavirus spreads on small droplets in the air when a person infected with Covid-19 breaths, coughs, sneezes or talks without wearing a mask. Therefore, keeping a physical social distance reduces any virus exposure and the risk of the virus to spread from person to person. We will ask you to judge the degree to which different distances in face to face situations can reduce exposure to the virus for persons who do not wear a mask.*”

The instruction continued with an example that introduced the problems about interpersonal distance and the instruction to the condition with exposure from a longer distance compared with a shorter distance included the following.

“*Assume that two persons are in a face to face conversation in 6 min standing 2 feet away from each other and one of them is infected by a Corona virus. If they had been further away from each other, for example, 6 feet the virus exposure would have been smaller.*

*What percentage of the airborne viruses reaching a person at 2 feet will reach a person at 6 feet? Please, answer with a percentage. Same* = *100%, Three quarters* = *75%, Half* = *50%, One quarter* = *25%, One tenth* = *10% *etc.*…*”.

The questionnaire can be found in the supplementary information. The problems were presented in blocks of 6 problems in each of the conditions: (a) from long to short distance (time constant), (b) short to long distance (time constant), (c) from short to long conversation time (distance constant) and (d) from long to short conversation time (distance constant). All blocks were presented to each participant with either distance or time presented first for half of the participants in a balanced design. Blocks with increasing distance or time were presented first for half of the participants in a balanced order. Hence, the design was 2 × 2 (distance/time presentation first × increasing/decreasing presentation first). The items in a block were presented in a unique random order to each participant. The distances and times in the problems are listed in Tables 1 and 2. Some general demographic questions were followed by a question about a participant’s average interpersonal distance during a conversation with a person not infected with the Corona virus and the shortest interpersonal distance that would make the participant feel sufficiently safe to start a face to face conversation with a Corona infected person. Last some demographic questions followed and also some questions that were not part of the present study.

**Table 1:** Medians and quartiles of exposure judgments and predictions according to VEM, n = 2.0 and the linear model with n = 1.0. Increasing and decreasing distance

	First distance (feet)	Second distance (feet)	Judged median exposure % (quartiles)	N	Predicted VEM n = 2.0	Predicted linear n = 1.0
Increasing distance						
1	4	5	75.0 (47.5–80.0)	96	64.0	80.0
2	2	6	30.0 (15.0–40.0)	95	11.0	33.0
3	4	6	60.0 (40.0–75.0)	96	44.4	66.0
4	2	5	40.0 (25.0–50.0)	95	16.0	40.0
5	5	6	80.0 (30.0–98.5)	96	69.4	83.3
6	2	4	50 (50.0–50.0)	96	25.0	50.0
Decreasing distance						
1	5	4	125 (100–175)	85	156	125
2	6	2	300 (300–500)	84	900	300
3	6	4	150 (150–200)	85	225	150
4	5	2	250 (215–500)	84	625	250
5	6	5	120 (100–150)	84	144	120
6	4	2	200 (200–313)	84	400	200

**Table 2:** Medians and quartiles of judgments and predictions according to a linear model. Decreasing and increasing time of conversation

	First time (minutes)	Second time (minutes)	Judged exposure	N	Predicted linear
Decreasing time					
2	10	1	10.0 (10–10)	96	10.0
3	6	1	17.0 (15–25)	96	17.0
4	10	3	30.0 (30.0–35.0)	96	30.0
5	3	1	33.0 (25.0–33.0)	96	33.0
6	10	6	60.0 (50.0–61.3)	96	60.0
Increasing time					
1	3	6	200 (200–200)	86	200
2	1	10	1000 (1000–1000)	90	1000
3	1	6	600 (500–600)	88	600
4	3	10	333 (300–500)	89	333
5	1	3	300 (200–300)	86	300
6	6	10	180 (165–400)	85	167

## Results

Eleven of the participants had been diagnosed with COVID-19. We will include also these participants in the group data analyses because the sample was too small for separate analyses. A number of participants who were asked to judge percentages greater than 100 in a decreasing distance condition (increasing exposure) gave judgments that were smaller than 100. In spite of a detailed instruction, they may have misunderstood the task so that they judged the increment in exposure instead of the total exposure after a change. Therefore, when a judgment was greater than 100 in an increasing distance condition (decreasing exposure) the judgment was coded as missing. Correspondingly, in an decreasing distance condition (increasing exposure) judgments that were smaller than 100 were coded as missing. However, the judgment distributions were still irregular with some very high and some very low values. This was reflected in high skewness (less than − 1.0 or greater than + 1.0) or moderate skewness (between ± 1.0 and ± 0.5) for the individual problems. This finding and the fact that we wanted to investigate the proportions of participants whose judgments deviated from estimated correct exposure values motivate the use of medians and quartiles in the results.

Table 1 gives medians and quartiles for each of the distance problems. In comparison with the VEM model with n = 2.0, the median participant overestimated exposure for increasing distance and underestimated exposure for decreasing distance. In other words, they judged the effects of a change of interpersonal distance on exposure to be smaller than the estimated objective effects.

For both increasing and decreasing distance separately, the differences between the median judgments and predictions of the VEM 2.0 model were significant in Wilcoxon Signed-Ranks tests, α = 0.05, N = 6 for both conditions. The numbers of observations were too small for a test based on normal distributions and therefore the z values are not reported. It is clear that the median judgments in Table 1 are closer to a linear function than to VEM with the exponent = 2.0. We found that a majority of the judgments (78% across increasing and decreasing distance problems) indicated that a majority of the participants did not realize the degree to which exposure changes when interpersonal distance changes. The results in Table 1 were visualized in Fig. 1, which shows systematic overestimations of exposure when a person moves away from an infected person (a) and underestimations of exposure when the person moves closer to the infected person (b).

Next, we investigated the perceived effect on exposure following changes of the duration of conversations. We predicted that the judgments should follow a linear function, that is VEM with an exponent = 1.0. Table 2 shows median judgments for decreasing and increasing time. The medians describe an almost perfect fit to a linear relation. This means that the participants were quite accurately sensitive to the effects on exposure following a change of the time of a conversation.

In summary, the results replicated the earlier findings with underestimations of the effects on virus exposure of changing distance in comparison with actual exposure [1]. Furthermore, the results showed that the median participant applied a correct linear model when she or he judged the effects of time of a conversation on virus exposure.

For each participant we computed the means across the increasing distance problems, M1 and across the decreasing distance problems, M2 separately. Increasing distance means that virus exposure decreases. Here, a relatively greater M1 means that a participant perceives *less decrease* of exposure following an increasing distance than participants who give relatively smaller judgments. Decreasing distance means that exposure increases. A relatively greater mean judgment means that a participant perceives *more increase* in exposure following a decreasing distance than participants who give relatively smaller judgments.

We asked our participants “What average distance do you keep from a person in a normal face to face conversation when no virus is around?” The mean response was 1.28 (SD = 0.52) meters. Then we asked “What is the shortest distance between you and a Corona virus infected person that would make you feel sufficiently safe to start a conversation of 3 min?” The mean was 3.51 (SD = 5.25) meters. Participants who require longer interpersonal distances can be assumed to perceive the effect of increasing distance to be smaller (that is why they want to be further away) than those who need a relatively shorter distance. We correlated the means M1 and M2 with safe distance. The Person correlation between M1 and safe distance was R (96) =  − 0.21 (− 0.39, − 0.01) p < 0.05 and between M2 and safe distance R(85) = 0.50 (0.32, 0.65) p < 0.001). Hence, the results gave some support for the asserted relationships.

## Discussion

As predicted, the studies showed that the effects on virus exposure of approaching and moving away from an infected person were underestimated. A linear instead of a curved relationship described the judgments, and hence explained most of the systematic underestimation bias. We used the exponent 2.0 in the VEM model to calculate the correct exposure values as a function of change of distance. This gives an underestimation in comparison with empirical studies of how quickly exposure changes with distance [9, 11, 22]. Therefore, when we reported that participants underestimated the change of exposure following a change of distance, this means that they were underestimating exposure in relation to already conservative objective estimates of change of exposure. Hence, the reported underestimations may be even greater than what we have reported. We did not treat long conversations in closed environments with the risk of ambient spread of virus aerosols. In such environments, no distance can protect a person from exposure and a face mask should be recommended. We have studied distancing and time in conversations with persons, none of whom wears a face mask. A face mask changes the air streams and emissions drastically [22] and this situation would be interesting to study in a new investigation. The normal interpersonal distance in a face to face conversation was 1.28 m. It was increased to 3.51 m to make the average participant feel safe when the other person was without a mask and infected with the Corona virus. It would be interesting to compare preferred safety distances for different viruses and use them as parameters in a new study.^1^ Then, it will be possible to see if a virus’ perceived safety distance affects the decreasing and increasing virus exposure functions. More contagious viruses, requiring longer safety distances, may induce different exposure judgment functions than less serious viruses. The prediction that the objective linear relationship between exposure and time would facilitate non-biased exposure judgments was confirmed.

The present results are relevant for people’s protective behaviors when there is an ongoing epidemic disease or pandemic. For example, people including health workers [23] may intuitively downplay the importance of keeping a longer interpersonal distance. Policy makers and politicians influenced by their intuitive understanding of virus exposure may underestimate the effects on the population level of recommending, for example, 2 instead of 1.5 m interpersonal distance. The present contribution studied perceptions of Corona virus exposure. However, a number of issues remain for further research. For instance, how does perceived exposure relate to infection risk perception? Do people assert linear relationships between exposure and infection risk? What protective role do they give a face mask [5, 24, 25]? Do people assume infection threshold effects? The Covid-19 pandemic illustrates the need of facts about intuitive perceptions, informed expert knowledge and communications about risks for informed decision making.

## Limitations

The results represent a sample of persons, who were available via Prolific, and therefore they do not represent a random selection of US citizens. But, the phenomenon studied is related to a common difficulty for people to handle curve linear relationships. The sample size did not permit any conclusions concerning subgroups of people, who may be more aware of the correct relationship between distance and virus exposure and less prone to make biased judgments. Another limitation is that the instruction did not explicitly specify the environmental context of the virus emitter and target person. This could have increased the variance of the judgments if the participants assumed spaces with significantly different air circulations. However, the present results for distance and judged exposure were the same as in the earlier study [1], in which, the environment was described as outside in calm weather or inside a big open well ventilated space indoors. We studied self reporting data, which may be biased, but we do not see any particular risk associated with the judgments reported here. However, possible inferences from judgments to behavior need to be investigated further. We studied Corona virus only, which limits possible conclusions about perceived exposure to other viruses with different degrees of contagiousness.

## Conclusions and recommendations

People quite drastically underestimate the increase in virus exposure following an approach to a virus infected person. They overestimate the remaining exposure after a move away from an infected person. Based on the information available to them, people develop their own mental models about causal and other relationships between different variables. Therefore, we should find out about these models and modify them if they are wrong or represent biased scientific facts. In the present case, this is relevant for peoples’ protective behaviors when there is an ongoing epidemic disease or pandemic. For public health reasons, the correct function connecting distance with virus exposure should be communicated to the general public to avoid deliberate violations of recommended interpersonal distances.

## Supplementary Information

Below is the link to the electronic supplementary material.

Supplementary file1 (DOC 111 KB)

## Data Availability

The data set generated and analyzed in this study is available from the author and also at Figshare via Stockholm University.https://su.figshare.com/articles/dataset/On_Interpersonal_Distance_Time_of_a_Conversation_and_Perceived_Virus_Exposure/20472165/1.

## Footnotes

1 This was suggested by one of the reviewers.

## Code availability

https://www.jamovi.org.
