# Supplementary material for: Perceived Corona virus exposure as a function of interpersonal distance and time of a conversation
Source: Discov Soc Sci Health. 2022 Dec 5;2(1):24. doi: 10.1007/s44155-022-00027-9 (PMC9734792; doi:10.1007/s44155-022-00027-9)
Supplement: Supplementary file 1 — Supplementary file1 (DOC 111 KB) [file 44155_2022_27_MOESM1_ESM.doc]

***Perceived corona virus exposure as a function of interpersonal distance and time of a conversation.***

*Discover Social Science and Health,*

*Ola Svenson, Stockholm University and Decision Research, osn@psychology.su.se*

**Demographic data sample of 96 participants**

| Age | M = 31 (SD=11.4) years |
| --- | --- |
| Gender | 48 women, 47 men,  1 unspecified |
| Diagnosed with Covid-19 | 11 |
| Member in extended family diagnosed with Covid-19 | 32 |
| Vaccinated participants | 77 |
| Have you avoided public spaces, crowds?  0= never 100 = always | M = 52.1 (SD = 35.8) |
| Have you canceled or postponed meetings with friends more often than before the pandemic?  0= never 100 = always | M = 37.4 (SD = 37.1) |
| Education | High school no graduate education (4 participants),  High school graduate (16) ,  Some college (26),  College graduate (39)  More than college graduate education (11). |

**Questionnaire**

intro **Introduction**

 As you probably know, the Coronavirus spreads on small droplets in the air when a person infected with Covid-19 breaths, coughs, sneezes or talks without wearing a mask. Therefore, keeping a physical social distance reduces virus exposure and the risk of the virus spreading from person to person.

 We will ask you to judge the degree to which different distances and durations of face-to-face interactions can reduce exposure to the virus for persons who do not wear a mask.

End of Block: Intro

Start of Block: Distance: increasing

| 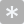 |
| --- |

Q1a
Assume that two people are in a face-to-face conversation for **6 minutes**, standing **2 feet apart** and one of them is infected with a Coronavirus. If they had been standing further away from each other (for example, 6 feet) the virus exposure ***would have been smaller***.
 
What percentage of the airborne viruses reaching a person at a distance of ***2 feet*** would reach a person at a distance of ***6 feet***?
  
Please answer with a number in the box below to indicate a percentage. Same = 100%, Three quarters = 75%, Half = 50%, One quarter = 25 %, One tenth = 10%, etc.
 
Remember that when a person *moves away* virus exposure *becomes smaller*.
   The percentage of airborne viruses will be:

%

________________________________________________________________

| Page Break |  |
| --- | --- |

Q1_6 Similarly, for each comparison below, what percentage of the airborne viruses will reach a person as they **move to a further distance**?

 Please answer with a number to indicate a percentage. Same = 100%, Three quarters = 75%, Half = 50%, One quarter = 25 %, One tenth = 10%, etc.

 Remember that when a person *moves away* virus exposure *becomes smaller*.

|  | Please enter responses below |
| --- | --- |
|  | 0-100% (1) |
| The percentage of viruses reaching the person from **5 feet compared to 4 feet** is: (1) |  |
| The percentage of viruses reaching the person from **6 feet compared to 2 feet** is: (2) |  |
| The percentage of viruses reaching the person from **6 feet compared to 4 feet** is: (3) |  |
| The percentage of viruses reaching the person from **5 feet compared to 2 feet** is: (4) |  |
| The percentage of viruses reaching the person from **6 feet compared to 5 feet** is: (5) |  |
| The percentage of viruses reaching the person from **4 feet compared to 2 feet** is: (6) |  |

End of Block: Distance: increasing

Start of Block: Distance: decreasing

Q13a
Assume that two people are in a face-to-face conversation for **6 minutes,** standing **5 feet** apart and one of them is infected with a coronavirus. If they had ***moved closer to each other*** (for example, to **2 feet**) the virus exposure ***would become greater***.
 
What percentage of the airborne viruses reaching a person at a distance of ***5 feet*** would reach a person at a distance of ***2 feet***?
 
Please, answer with a percentage. Same = 100%, twice = 200%, 5 times = 500%, 10 times = 1 000%, 100 times = 10 000%, etc.
 Remember that when a person *moves closer* virus exposure *becomes greater.*

 The percentage is:

%

________________________________________________________________

| Page Break |  |
| --- | --- |

Q13_18 Similarly, for each comparison below, what percentage of the airborne viruses will reach a person as they ***move closer***?

 Please, answer with a percentage. Same = 100%, twice = 200%, 5 times = 500%, 10 times = 1 000%, 100 times = 10 000%, etc.

 Remember that when a person *moves closer* virus exposure *becomes greater.*

|  | Please enter responses below |
| --- | --- |
|  | _ (1) |
| The percentage of viruses reaching the person from **4 feet compared to 5 feet** is (1) |  |
| The percentage of viruses reaching the person from **2 feet compared to 6 feet** is (2) |  |
| The percentage of viruses reaching the person from **4 feet compared to 6 feet** is (3) |  |
| The percentage of viruses reaching the person from **2 feet compared to 5 feet** is (4) |  |
| The percentage of viruses reaching the person from **5 feet compared to 6 feet** is (5) |  |
| The percentage of viruses reaching the person from **2 feet compared to 4 feet** is (6) |  |

End of Block: Distance: decreasing

Start of Block: Time: decreasing

Q7a
Assume that two people are in a face-to-face conversation for 6 minutes and one of them is infected with a coronavirus. If this conversation ***became shorter***, then the virus exposure would ***become smaller***.
 
What percentage of the airborne viruses reaching a person during a shorter conversation of ***2 minutes*** at a distance of ***3 feet*** will reach a person compared with the exposure of **6 minutes** at the same distance?
 
Please, answer with a percentage. Same = 100%, Three quarters = 75%, Half = 50%, One quarter = 25 %, One tenth = 10% etc.
 
Remember that when *time is shorter* exposure *becomes smaller*.
 
The percentage of airborne viruses will be:  
%

________________________________________________________________

| Page Break |  |
| --- | --- |

Q7_12 Similarly, for each comparison below, what percentage of the airborne viruses will reach a person as the ***conversation becomes shorter***?

 Please answer with a number to indicate a percentage. Same = 100%, Three quarters = 75%, Half = 50%, One quarter = 25 %, One tenth = 10%, etc.

 Remember that when *time is shorter* exposure *becomes smaller*.

|  | Please enter responses below |
| --- | --- |
|  | 0-100% (1) |
| The percentage of viruses reaching the person during **3 min compared to 6 min** is: (1) |  |
| The percentage of viruses reaching the person during **1 min compared to 10 min** is: (2) |  |
| The percentage of viruses reaching the person during **1 min compared to 6 min** is: (3) |  |
| The percentage of viruses reaching the person during **3 min compared to 10 min** is: (4) |  |
| The percentage of viruses reaching the person during **1 min compared to 3 min** is: (5) |  |
| The percentage of viruses reaching the person during **6 min compared to 10** **min** is: (6) |  |

End of Block: Time: decreasing

Start of Block: Time: increasing

Q19a
Assume that two people are in a face-to-face conversation for 2 minutes and one of them is infected with a coronavirus. If this conversation ***became longer***, then, the virus exposure would ***become greater***.
 
What percentage of the airborne virus reaching a person during a longer conversation of ***6 minutes*** at a distance of 5 feet will reach a person compared with the exposure of ***2 minutes*** at the same distance?
 
Please, answer with a percentage. Same = 100%, twice = 200%, 5 times = 500%, 10 times = 1 000%, 100 times = 10 000% etc.
 
Remember that when *time is longer* exposure *becomes greater*.
 
The percentage of airborne viruses will be:  
%

________________________________________________________________

| Page Break |  |
| --- | --- |

Q19_24 Similarly, for each comparison below, what percentage of the airborne viruses will reach a person as the ***conversation becomes longer***?
  
Please answer with a percentage. Same = 100%, twice = 200%, 5 times = 500%, 10 times = 1 000%, 100 times = 10 000% etc.
 
Remember that when *time is longer* exposure *becomes greater*.

|  | Please enter responses below |
| --- | --- |
|  | - (1) |
| The percentage of viruses reaching the person during **6 min compared to 3 min** is (1) |  |
| The percentage of viruses reaching the person during **10 min compared to 1 min** is (2) |  |
| The percentage of viruses reaching the person during **6 min compared to 1 min** is (3) |  |
| The percentage of viruses reaching the person during **10 min compared to 3 min** is (4) |  |
| The percentage of viruses reaching the person during **3 min compared to 1 min** is (5) |  |
| The percentage of viruses reaching the person during **10 min compared to 6 min** is (6) |  |

End of Block: Time: increasing

Start of Block: Practices

Q25
You have answered questions about inter-personal distance and virus exposure. What average distance do you keep from a person in a normal face-to-face conversation **when no virus is around**?
 
Distance kept (feet):

________________________________________________________________

Q26a
If you were infected with a new Coronavirus, to what extent do you think that this would have been caused by just you being unlucky or by your own poor protective behavior?

|  | 0 = Not at all | 100 = Only poor luck |
| --- | --- | --- |

|  | 0 | 10 | 20 | 30 | 40 | 50 | 60 | 70 | 80 | 90 | 100 |
| --- | --- | --- | --- | --- | --- | --- | --- | --- | --- | --- | --- |

| Due to my poor luck () | 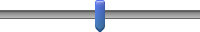 |
| --- | --- |

Q26b

|  | 0 = Not at all | 100 = Only poor protective behavior |
| --- | --- | --- |

|  | 0 | 10 | 20 | 30 | 40 | 50 | 60 | 70 | 80 | 90 | 100 |
| --- | --- | --- | --- | --- | --- | --- | --- | --- | --- | --- | --- |

| Due to my poor protective behavior () | 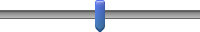 |
| --- | --- |

| Page Break |  |
| --- | --- |

Q27
What is the shortest distance between you and a Coronavirus-infected person that would make you feel sufficiently safe to start a conversation of 3 minutes?


Distance in feet:

________________________________________________________________

Q28 How worried have you been over your own personal risk of becoming sick with COVID-19 during the pandemic?

|  | 0 = Not at all | 100 = Maximum |
| --- | --- | --- |

|  | 0 | 10 | 20 | 30 | 40 | 50 | 60 | 70 | 80 | 90 | 100 |
| --- | --- | --- | --- | --- | --- | --- | --- | --- | --- | --- | --- |

| 1 () | 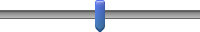 |
| --- | --- |

Q29 In general, how worried are you over things that may go wrong in your life?

|  | 0 = Not at all | 100 = Maximum |
| --- | --- | --- |

|  | 0 | 10 | 20 | 30 | 40 | 50 | 60 | 70 | 80 | 90 | 100 |
| --- | --- | --- | --- | --- | --- | --- | --- | --- | --- | --- | --- |

| 1 () | 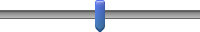 |
| --- | --- |

Q30 If you always follow the advise of keeping distance from other people, to what degree do you think that this behavior can protect you from being infected by a Coronavirus, assuming no masks are used?

|  | 0 = Not at all | 100 = Maximum |
| --- | --- | --- |

|  | 0 | 10 | 20 | 30 | 40 | 50 | 60 | 70 | 80 | 90 | 100 |
| --- | --- | --- | --- | --- | --- | --- | --- | --- | --- | --- | --- |

| 1 () | 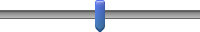 |
| --- | --- |

| Page Break |  |
| --- | --- |

Q31 How old are you? (years)

________________________________________________________________

Q32 Which option best describes your gender?

- Male (1)
- Female (2)
- Something else (3) ________________________________________________

| Page Break |  |
| --- | --- |

Q33 Have you been diagnosed with COVID-19 by a test or by a doctor?

- Yes (1)
- No (2)

Display This Question:

If Have you been diagnosed with COVID-19 by a test or by a doctor? = Yes

Q34a Did you become sick from the COVID-19 infection?

- Yes (1)
- No (2)

Display This Question:

If Did you become sick from the COVID-19 infection? = Yes

| 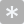 |
| --- |

Q34b
How many days were you sick?

________________________________________________________________

| Page Break |  |
| --- | --- |

Q35 Has anyone in your extended family been diagnosed with COVID-19?

- Yes (1)
- No (2)

| 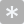 |
| --- |

Q36
If a person in your close family was sick, for how many days (if more than one person, the person who was most sick)?


- Number of days:

________________________________________________________________

| Page Break |  |
| --- | --- |

Q37 Are you vaccinated against COVID-19?

- Yes (1)
- No or only one dose (if vaccine requires two doses, e.g., Pfizer, Moderna) (2)

Display This Question:

If Are you vaccinated against COVID-19? = Yes

Q37_vax_mm In which month of 2021 did you receive your final dose?

- January (1)
- February (2)
- March (3)
- April (4)
- May (5)
- June (6)
- July (7)
- August (8)
- September (9)
- October (10)

| Page Break |  |
| --- | --- |

Q38
Assume a new Coronavirus epidemic occurred and no vaccine was available. Compared to the average person like yourself, how likely do think that it is that you would become sick?


(Much less risk = 1, same risk as average person = 50, much greater risk =100)


My risk would be (scale 1-100):

________________________________________________________________

Q39
If you should be infected with a new Coronavirus without a vaccine, what is the risk that you would die from the infection?


(No risk = 0 , certain that I would die = 100)

________________________________________________________________

End of Block: Practices

Start of Block: Block 4

Q40_41 Which of the following have you done during the **last seven days** of the present Coronavirus pandemic?

|  | 0 = Never | 100 = Always |
| --- | --- | --- |

|  | 0 | 10 | 20 | 30 | 40 | 50 | 60 | 70 | 80 | 90 | 100 |
| --- | --- | --- | --- | --- | --- | --- | --- | --- | --- | --- | --- |

| Avoided public spaces, gatherings or crowds more often than before the pandemic? () | 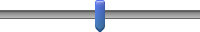 |
| --- | --- |
| Canceled or postponed meetings with friends more often than before the pandemic? () | 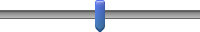 |

End of Block: Block 4

Start of Block: Block 5

block_instr We will now present some additional problems. Please, solve them as correctly as you can without using a calculator.

Q42 A bat and a ball cost $110 in total. The bat costs $100 more than the ball. How much does the ball cost?

 - The cost of the ball in dollars:

________________________________________________________________

Q43 If it takes 5 machines 5 minutes to make 5 widgets, how long would it take 100 machines to make 100 widgets?

 - Number of minutes:

________________________________________________________________

Q44 In a lake, there is a patch of lily pads. Every day, the patch doubles in size. If it takes 48 days for the patch to cover the entire lake, how long would it take for the patch to cover half of the lake?

 - Number of days:

________________________________________________________________

End of Block: Block 5

Start of Block: Education

Q45 Please indicate your highest level of education

- 8th grade or less (1)
- High school, no graduate (2)
- High school graduate (3)
- Vocational (4)
- Some college (5)
- College graduate (6)
- More than college graduate (specify: MA/MS, JD, MD, PhD or other) (7) ________________________________________________

End of Block: Education

Start of Block: Attention Check

Q46 Most modern theories of decision making recognize the fact that decisions do not take place in a vacuum. Individual preferences and knowledge, along with situational variables can greatly impact the decision process. In order to facilitate our research on decision making we are interested in knowing certain factors about you, the decision maker. Specifically, we are interested in whether you actually take the time to read the directions; if not, then some of our manipulations that rely on changes in the instructions will be ineffective. So, in order to demonstrate that you have read the instructions simply type in “I read the instructions” in the “Other” space. Thank you very much.

- Watching athletics (1)
- Electronic games (2)
- Needlework (3)
- Participate in athletics (4)
- Board or Card games (5)
- Gardening (6)
- Reading (7)
- Attending Cultural Events (8)
- Clubbing (9)
- Watching movies (10)
- Religious Activites (11)
- Travel (12)
- Hiking/Camoing (13)
- Cooking (14)
- Other: (15) ________________________________________________
